# The Role of Bone Morphogenetic Protein 9 in Nonalcoholic Fatty Liver Disease in Mice

**DOI:** 10.3389/fphar.2020.605967

**Published:** 2021-02-02

**Authors:** Qin-Juan Sun, Ling-Yan Cai, Jie Jian, Ya-Lu Cui, Chen-Kai Huang, Shu-Qing Liu, Jin-Lai Lu, Wei Wang, Xin Zeng, Lan Zhong

**Affiliations:** ^1^ Department of Gastroenterology, Shanghai East Hospital, Tongji University School of Medicine, Shanghai, China; ^2^ Department of Gastroenterology, Changzheng Hospital, Second Military Medical University, Shanghai, China

**Keywords:** BMP9, NAFLD, hepatic steatosis, glucose metabolism, inflammatory response, RNA-seq, ATAC-seq

## Abstract

**Background and Aims:** It’s reported that bone morphogenetic protein 9 (BMP9) played an important role in lipid and glucose metabolism, but the role of BMP9 in nonalcoholic fatty liver disease (NAFLD) is unclear. Here, we evaluated the therapeutic efficacy of recombined BMP9 in NAFLD mice and investigated the potential mechanism.

**Methods:** The effects of recombinant BMP9 on NAFLD were assessed in HFD-induced NAFLD mice. C57BL/6 mice were administrated with high-fat diet (HFD) for 12 weeks. In the last 4 weeks, mice were treated with PBS or recombined BMP9 once daily. Insulin sensitivity was evaluated by glucose tolerance test (GTT) and insulin tolerance test (ITT) at the end of the 12th week. Then NAFLD related indicators were assessed by a variety of biological methods, including histology, western blotting, real-time PCR, RNA-seq and assay for transposase-accessible chromatin using sequencing (ATAC-seq) analyses.

**Results:** BMP9 reduced obesity, improved glucose metabolism, alleviated hepatic steatosis and decreased liver macrophages infiltration in HFD mice. RNA-seq showed that Cers6, Cidea, Fabp4 involved in lipid and glucose metabolism and Fos, Ccl2, Tlr1 involved in inflammatory response downregulated significantly after BMP9 treatment in HFD mouse liver. ATAC-seq showed that chromatin accessibility on promoters of Cers6, Fabp4, Ccl2 and Fos decreased after BMP9 treatment in HFD mouse liver. KEGG pathway analysis of dysregulated genes in RNA-seq and integration of RNA-seq and ATAC-seq showed that TNF signaling pathway and Toll-like receptor signaling pathway decreased in BMP9 treated HFD mouse liver.

**Conclusion:** Our data revealed that BMP9 might alleviate NAFLD via improving glucose and lipid metabolism, decreasing inflammatory response and reshaping chromatin accessibility in HFD mouse liver. BMP9 downregulate genes related to lipid metabolism, glucose metabolism and inflammation expression, at least partially via decreasing promoter chromatin accessibility of Cers6, Fabp4, Fos and Tlr1. BMP9 may also reduce the expression of liver Ccl2, thereby changing the number or composition of liver macrophages, and ultimately reducing liver inflammation. The effect of BMP9 on NAFLD might be all-round, and not limit to lipid and glucose metabolism. Therefore, the underlying mechanism needs to be studied in detail further.

## Introduction

Non-alcoholic fatty liver disease (NAFLD) is the most common chronic liver disease worldwide. It is associated with clinical states, such as obesity, insulin resistance, and type 2 diabetes. NAFLD encompasses a wide range of liver diseases, ranging from simple steatosis, nonalcoholic steatohepatitis, to fibrosis and finally cirrhosis and hepatocellular carcinoma (Eslam et al., 2018). Most of the pathological processes are histologically characterized by hepatocyte steatosis, hepatocellular ballooning, lobular inflammation and fibrosis (Baffy et al., 2012). According to the “multi-hits hypothesis”, steatosis, lipotoxicity and inflammation promote the pathological progression of NAFLD (Cobbina et al., 2017). Insulin resistance further aggravates lipid accumulation by causing an imbalance of lipid metabolism (Straub et al., 2010; Takaki et al., 2013). However, the underlying pathogenesis leading to NAFLD progression has not been completely elucidated until now. Alleviating steatosis, inflammation and insulin resistance may provide beneficial effects for the treatment of NAFLD.

Bone Morphogenetic Proteins (BMPs) are cytokines that belong to the TGF-β superfamily (Herrera et al., 2014) and are mainly associated with fat differentiation and energy balance (Tobin and Celeste, 2006; Schulz and Tseng, 2009). BMP9, a member of this family, is primarily expressed in the liver and presents in the serum at bioactive concentrations (Song et al., 1995). BMP9 binds to a hetero-tetrameric receptor complex comprised of Type I and Type II receptors, initiating downstream signaling cascades. The canonical signaling pathway involves the phosphorylation and subsequent activation of Smad1, 5 and 9. Then, activated Smad1/5/9 partners with Smad4 to act as a transcription factor to regulate target gene expression (Gomez-Puerto et al., 2019). It has been reported that hepatic stellate cells are the main source and the target cells of BMP9 (Breitkopf-Heinlein et al., 2017). BMP9 also binds to specific receptors on hepatocytes and induces proliferation of cultured primary rat hepatocytes and HepG2 cells (Song et al., 1995). Several studies have demonstrated BMP9 is one of factors that may induce liver fibrosis by potentially regulating the process of fibrosis (Bi and Ge, 2014). Li et al. (2018) found that higher BMP9 levels accompanied advanced stages of liver fibrosis in liver fibrotic patients, recombinant Bmp9 overexpression accelerated liver fibrosis, and adenovirus-mediated Bmp9 knockdown attenuated liver fibrogenesis in mouse models. The research of Breitkopf-Heinlein et al. (Breitkopf-Heinlein et al., 2017) also revealed that lack of BMP-9 *in vivo* ameliorated liver fibrosis upon repeated injections of CCl4. However, John et al. (2019) revealed that BMP9 expression had a downregulation rather than upregulation in a cirrhotic liver. The role of BMP9 on liver fibrosis still controversial. Furthermore, the effect of BMP9 on hepatocyte steatosis remains to be elucidated.

More recently, BMP9 has been demonstrated to interact with the enzymes associated with energy metabolism *in vivo* (Miller et al., 2000; Chen et al., 2003; Caperuto et al., 2008). The expression of BMP9 decreased in type 2 diabetes and NASH patients (John et al., 2019; Yang et al., 2019). Rats treated with anti-BMP9 antibodies led to impaired glucose tolerance and insulin resistance (IR). In addition, adenovirus-mediated BMP9 overexpression reduced weight gain and blood glucose levels and improved insulin resistance in high-fat diet (HFD)-fed mice (Kuo et al., 2014; Yang et al., 2019). Yang et al. infected HepG2 cells and mouse primary hepatocytes with either Ad-BMP9 or Ad-GFP and found that Ad-BMP9 treatment could decreased body weight, food intake and liver weight/body weight, decrease liver steatosis, inhibited the expression of genes related to fatty acid synthesis (Yang et al., 2019). Mechanistically, they found that BMP9 could inhibit the expression of sterol regulatory element binding protein-1 (SREBP1) through decreased transcriptional activity of liver X receptor response element 1. These findings suggest that BMP9 might play a key role in lipid and glucose metabolism and may be used for NAFLD therapy. However, the exact efficacy and mechanism of BMP9 in the treatment of NAFLD remain unclear.

Chromatin is a dynamic central regulator of transcription. The accessibility of chromatin is important for modulating gene expression during normal development (Stergachis et al., 2013; Wu et al., 2016) and results in distinct cellular phenotypes (Shen et al., 2012). Open chromatin regions in transcription components, such as gene promoters and enhancers, activate gene transcription. Closed chromatin regions impair the accessibility of promoters and enhancers to transcription factors, causing changes in gene expression. Alterations in chromatin accessibility and gene expression have been reported in NAFLD mice induced by a high fat diet (Leung et al., 2014). As BMP9 is an important regulator of energy homeostasis, its role in regulating chromatin accessibility of HFD-fed mice needs to be evaluated.

In this study, we evaluated the therapeutic efficacy of recombinant BMP9 in NAFLD mice and found that exogenous supplementation of BMP9 reduced obesity and hepatic steatosis and improved glucose metabolism. RNA-seq showed that BMP9 downregulated the expression of lipid metabolism and inflammation genes. We also analysed chromatin accessibility and identified putative transcription factors (TFs) by using ATAC-seq. Interestingly, the integration analysis of RNA-seq and ATAC-seq suggested that BMP9 downregulates inflammatory responses in NAFLD.

## Materials and Methods

### Animals and Treatments

Six-week-old male C57BL/6 mice (16–18 g) were purchased from Shanghai Jihui Laboratory Animal Co., Ltd. (Shanghai, China). The animals were maintained in the experimental animal centre of the Second Military Medical University. All animal experiments were approved by the Scientific Investigation Board of the Second Military Medical University. Mice were fed an HFD diet (protein, 14.1%; fat, 60%; carbohydrates, 25.9%; Trophic) and randomly divided into two groups: the HFD group (*n* = 8) and the HFD+BMP9 group (*n* = 8). The control group (Control, *n* = 6) was fed a normal chow diet and treated with PBS. The HFD group was fed an HFD and administered PBS. The HFD+BMP9 group was fed an HFD and administered recombinant murine BMP9 (PEPROTECT, Lot#:553204) at 200 ng/mouse. All mice were treated with an intraperitoneal injection of PBS or BMP9 once daily for 4 weeks. After 12 weeks, all mice were sacrificed, and liver, serum and visceral adipose tissue (VAT) were collected.

### Histological Analysis

Liver and VAT were fixed in 10% formalin and 4% paraformaldehyde and then embedded in paraffin or frozen following standard procedures. Liver sections were stained with haematoxylin and eosin (H&E), Sirius red and oil red O. The extent of NAFLD was estimated by the NAFLD activity score (NAS). The intensity of steatosis was calculated as the percentage of the positive area of Oil red O staining in the corresponding field of liver tissue using image analysis software (IMAGE-PRO Plus 6.0; Media Cybernetics, Rockville, MD, United States).

### Immunohistochemical Staining

Immunohistochemical staining was performed on 4 μm-thick paraffin sections of tissues fixed in buffered formalin. Sections were deparaffinized in xylene and rehydrated in graded alcohols. Endogenous peroxidase was blocked by 3% H_2_O_2_ followed by antigen retrieval. Slides were incubated with primary antibodies overnight at 4°C and incubated with a secondary antibody at room temperature for 60 min. The following primary antibodies were used: BMP9 (sc514211, Santa Cruz) and F4/80 (70,076, Cell Signaling Technologies). The staining was developed using an EnVision Detection Rabbit/Mouse Kit (GK500710, GeneTech, Shanghai, China).

### Determination of Triglyceride (TG) and Cholesterol (CHOL)

The contents of TG and CHOL in mouse livers were measured using a commercial kit (TG: A0-10017; CHOL: A111-2-1, Jiancheng company, Nanjing, China).

### Quantitative Real-Time PCR

Total RNA was extracted from liver tissues with TRIzol (Invitrogen, Carlsbad, CA, United States). Transcript levels were detected via real-time reverse transcription polymerase chain reaction (RT-PCR) with a SYBR Green PCR Kit (Takara, Tokyo, Japan). The primers are listed in Supplementary Table S1.

### Western Blot Analysis

Tissues and cells were lysed in lysis buffer (125 mM Tris-HCl pH 6.8, 25% glycerol, 5% SDS) supplemented with protease inhibitor (Roche, Switzerland). Lysates were separated on sodium dodecyl sulfate polyacrylamide gels and transferred to methanol-activated nitrocellulose membranes (HAHY00010; Millipore, Merck KGaA, Darmstadt, Germany). Membranes were blocked in PBS containing 1% Tween-20 and 5% milk for 1 h and incubated with primary antibodies overnight at 4°C. After 1 h of incubation with donkey anti-mouse or donkey anti-rabbit secondary antibodies (IRDye 800; LI-COR Biosciences, Lincoln, NE, United States), signals were imaged using an Odyssey infrared imaging system (LI-COR Biosciences) at a wavelength of 700/800 nm. The primary antibodies used were: SREBP1 (ab96777, Abcam; sc-557036, Santacruz), phospho-Akt (4060, Cell Signaling Technologies), Akt (40D4, Cell Signaling Technologies), and GAPDH (YM3029, Immunoway).

### Intra-Peritoneal Glucose Test (IPGTT) and Intra-peritoneal Insulin Tolerance Test (IPITT)

The glucose tolerance test measures the clearance of an intraperitoneally injected glucose load from the body. IPGTT and IPITT are used to detect disturbances in glucose metabolism. Mice were fasted for 16 h for the IPGTT and 6 h for the IPITT, and fasted blood glucose levels (FBG) were determined before a solution of glucose ((1.2 g/kg) or insulin (0.75 mU/kg) was administered by intra-peritoneal (IP) injection. Subsequently, the tail vein blood glucose level was measured at different time points (30, 60, 90, and 120 min). Blood glucose concentrations were measured with an ACCU-CHEK Glucometer (Roche, Basel, Switzerland).

### Biochemical Assays

Serum biochemical parameters were measured with an automatic analyser in the Clinical Immunology department in Eastern Hepatobiliary Surgery Institute.

### RNA Sequencing (RNA-seq)

Total RNA was isolated from liver samples using the RNeasy mini kit (Qiagen, Germany). Paired-end libraries were synthesized using the TruSeq® RNA Sample Preparation Kit (Illumina, USA) following the TruSeq® RNA Sample Preparation Guide. Library construction and sequencing were performed at Shanghai Biotechnology Corporation. The fold-changes were estimated according to the FPKM in each sample. Significantly differentially expressed genes were selected using the following filter criteria: *p*-value ≤ 0.05, ∣Log2FC∣ > 1. The data are accessible at the NCBI-Bio Project under accession number PRJNA644055.

### Assay for Transposase-Accessible Chromatin Using Sequencing (ATAC-seq)

ATAC-seq was performed as previously described with fresh liver tissues (Buenrostro et al., 2015). MACS2 (2.1.1) was used to call peaks, and an initial threshold was defined as *p* < 1*10^−5^. The pair-wise Spearman correlation between any pair of ATAC-seq samples was calculated based on read counts/signals on merged ATAC-seq peaks from all samples. ATAC-seq peaks were annotated using Homer’s annotate Peaks.pl. The protein-binding motifs were identified using HOMER software (http://homer.ucsd.Edu/homer/). Three biological replicates were used. The data are accessible at the NCBI-Bio Project under accession number PRJNA644146.

### Statistical Analysis

GraphPad Prism 7.0 software (GraphPad software, La Jolla, CA) was used to perform data analyses to evaluate the statistical significance of the differences. Unpaired Student’s t-test was performed to compare between groups. All data are expressed as the means ± SEM. Statistical significance was set at *p ≤ 0.05, **p ≤ 0.01, and ***p ≤ 0.001. Differences with *p-*values < 0.05 were considered statistically significant.

## Results

### BMP9 Expression Is Downregulated in HFD-Induced NAFLD in Mice

**FIGURE 1:**
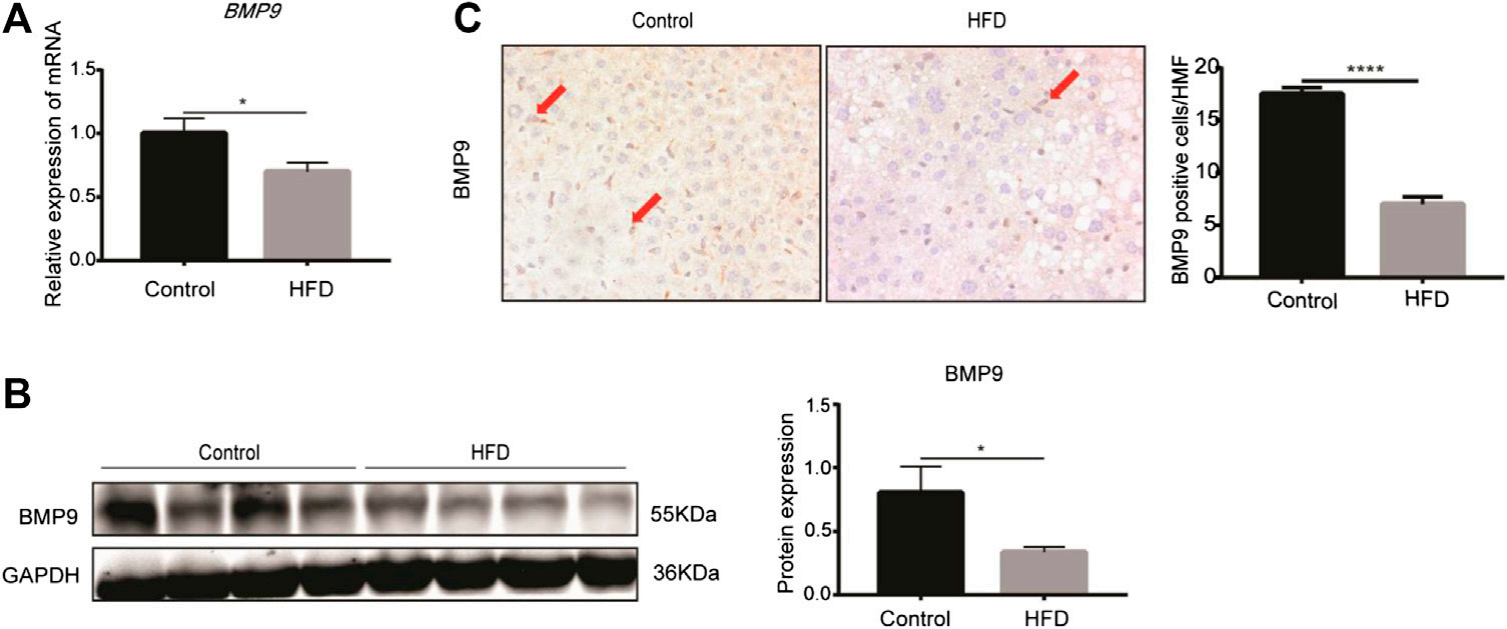
BMP9 expression is downregulated in HFD-fed mice (Pictures in Figure 1C were changed. BMP9 positive cells were counted in different groups). **(A)** The mRNA expression of BMP9 in HFD-fed mice was downregulated. **(B)** Western blot showed that protein expression of BMP9 in HFD-fed mice was downregulated. **(C)** IHC staining results showed that expression of BMP9 decreased in HFD mice compared to control mice. BMP9 was expressed mainly in non-parenchymal cells. Control, *n* = 6; HFD, *n* = 7. The data are presented as the means ± SEM. *p < 0.05.

The levels of BMP9 mRNA and protein expression were examined in the fatty livers of HFD mice. RT-PCR showed that the mRNA levels of BMP9 were downregulated in HFD mice compared to control mice (Figure 1A). Western blot also showed that BMP9 protein expression was decreased in fatty livers (Figure 1B). Moreover, immunostaining showed much lighter staining of BMP9 in non-parenchymal cells of the fatty livers than the control livers (Figure 1C). All these data confirmed that the expression of BMP9 is decreased in HFD-induced NAFLD mouse livers.

### BMP9 Reduces HFD-Induced Obesity in Mice

**FIGURE 2:**
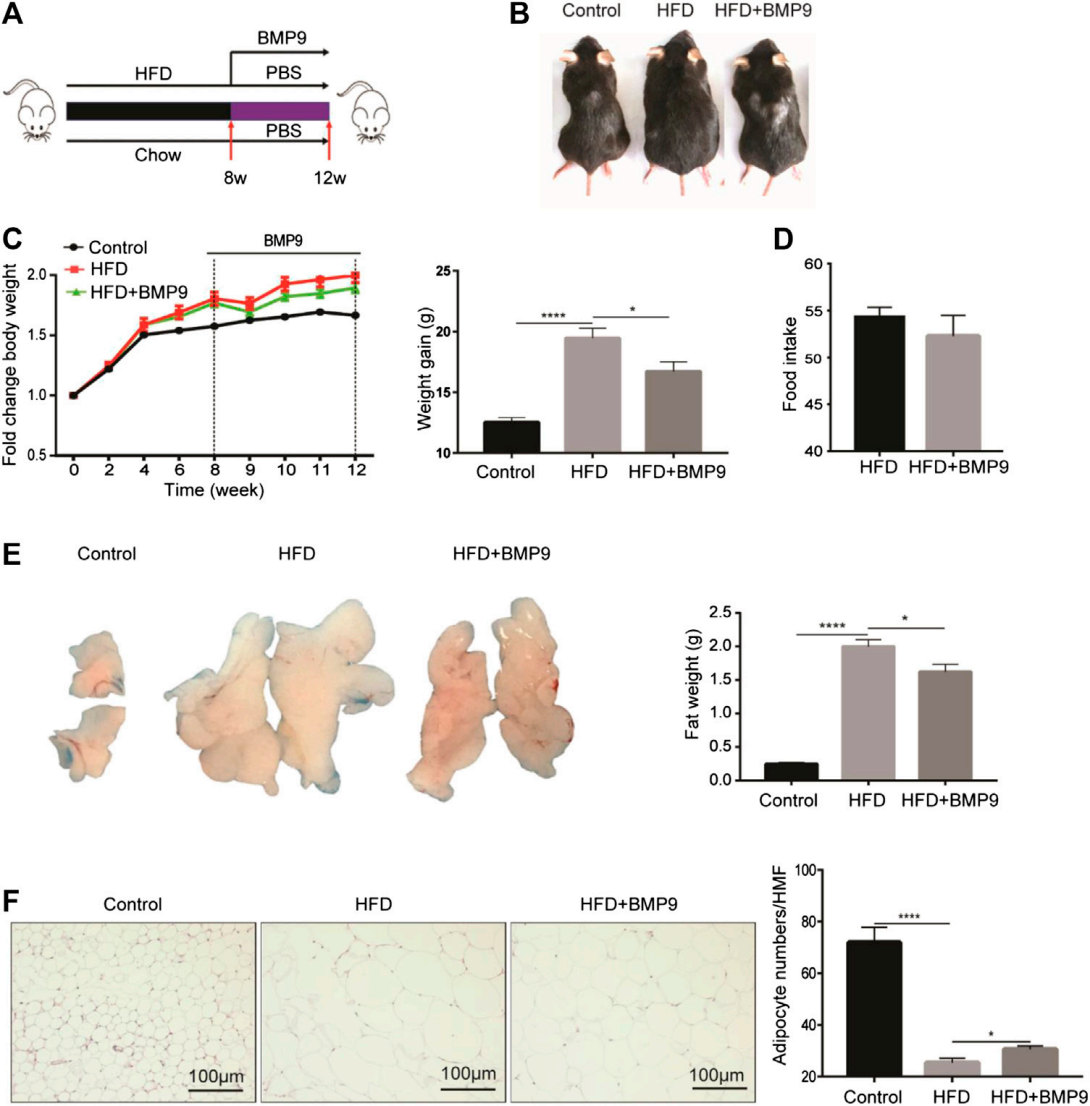
BMP9 reduces HFD-induced obesity in mice (Figure 2C Increasing body weight was changed as weight gain). **(A)** Experimental flow chart. Male mice were fed an HFD (containing 60% fat) for 8 weeks and administered PBS or recombinant BMP9 200 ng/mice/day via intraperitoneal injection for another 4 weeks. **(B)** Representative images of mouse in different groups. **(C)** Body weight was measured at different timepoints. Weight gain was measured from week 0–12. **(D)** Food intake monitored over the last month. **(E)** The appearance and weight of VAT. **(F)** HE staining of VAT (original magnification, ×200) and adipocyte numbers/HMF of VAT in different groups. HMF: High magnification field. The data are presented as the means ± SEM. Control, *n* = 6; HFD and HFD+BMP9, *n* = 8 respectively. *p < 0.05, ****p < 0.0001.

As mentioned above, the expression of BMP9 was downregulated in HFD-induced fatty liver. Therefore, we further investigated the role of exogenous BMP9 supplementation on NAFLD by intraperitoneal injection of BMP9 to HFD mice for 4 weeks (Figure 2A). Morphological observations revealed that HFD mice treated with BMP9 were smaller than PBS-treated mice (Figure 2B), and compared to PBS-treated mice, BMP9-treated mice gained less weight (Figure 2C). In despite of no significant differences in food intake were found between BMP9- and PBS-treated HFD mice (Figure 2D), BMP9 obviously reduced the visceral adipose tissue (VAT) weight of HFD mice (Figure 2E). Meanwhile, the volume of VAT in HFD mice was larger than that in BMP9-treated mice. Histological staining and analysis of adipose tissue showed that adipocyte size of the BMP9-treated mice was smaller than that of the HFD group (Figure 2F). ×400 magnification was used to observe HE staining of fat tissue. Take five different fields for each tissue to count cells under microscope. Finally take the average. The difference in the number of cells between the last average adipocyte numbers of each groups was compared. These results suggested that BMP9 reduces HFD-induced obesity in mice.

### BMP9 Treatment Attenuates Hepatic Steatosis and Macrophage Infiltration in HFD-Induced NAFLD in Mice

**FIGURE 3:**
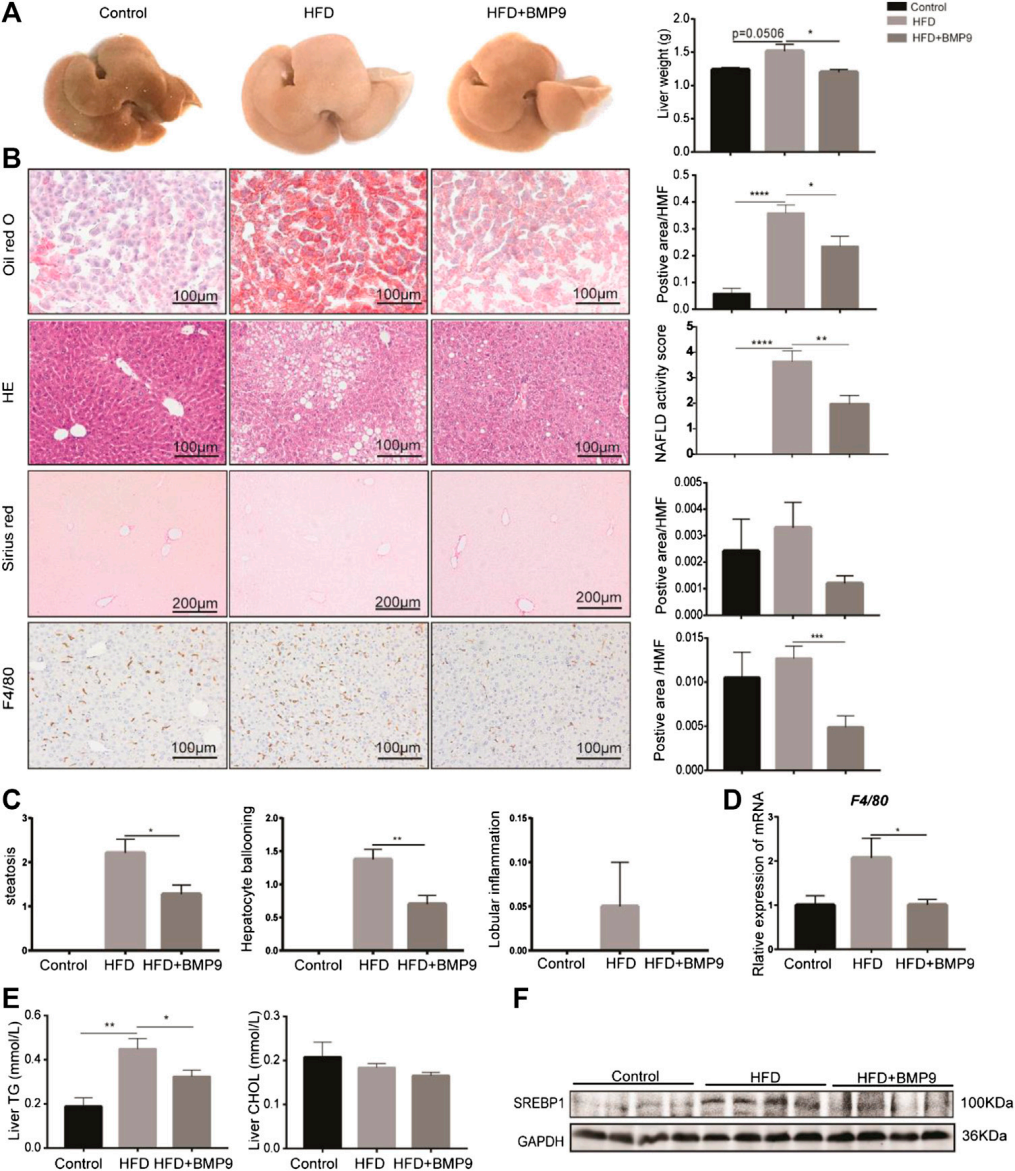
BMP9 treatment attenuates hepatic steatosis in HFD mouse livers. **(A)** The appearance of the liver and liver weight difference among the three groups. **(B)** Oil red o staining, HE, sirius red staining of the liver and F4/80 IHC staining in the different groups (original magnification, ×200 or ×100). **(C)** Histological scoring of steatosis, hepatocyte ballooning and lobular inflammation. **(D)** The mRNA expression of F4/80 in mouse livers. **(E)** BMP9 modulated TG but not CHOL levels in the liver. **(F)** BMP9 downregulated SREBP1 protein expression in the liver. The data are presented as the means ± SEM. Control, *n* = 6; HFD and HFD+BMP9, *n* = 8 respectively. *p < 0.05, **p < 0.01, ***p < 0.001, ****p < 0.0001.

Grossly, the livers of HFD mice appeared significantly paler than those of the normal group, while the colour of livers from the BMP9-treated group was recovered (Figure 3A). Furthermore, the liver weight was decreased in BMP9-treated HFD mice compared to HFD mice. Histologically, BMP9 significantly alleviated hepatic steatosis and hepatocyte ballooning in HFD-fed mice (Figure 3C), and the NAS was clearly decreased in BMP9-treated mice compared to untreated mice. Although the levels of serum triglyceride, cholesterol, alanine aminotransferase and aspartate aminotransferase were not significantly different regardless of BMP9 treatment (Supplementary Figure S1A–D), BMP9 reduced liver triglyceride levels in HFD-fed mice (Figure 3E). Moreover, the protein level of SREBP1, an important TF of lipogenesis, was downregulated in BMP9-treated mice (Figure 3F).

It has been reported that macrophages play a central role in the progression of NAFLD^[25]^. We next investigated the overall liver infiltration of macrophages. F4/80, a marker of macrophages, was observed in mouse livers. Immunohistochemistry analysis showed that BMP9 treatment reduced the population of F4/80-positive cells in HFD-fed mice (Figure 3B). Moreover, the mRNA expression of F4/80 was also significantly decreased in BMP9-treated mice compared to HFD mice (Figure 3D). Collectively, these data suggested that BMP9 treatment attenuates hepatic steatosis and macrophage infiltration in NAFLD in mice.

### BMP9 Modulates Glucose Metabolism in HFD-Induced NAFLD in Mice

**FIGURE 4:**
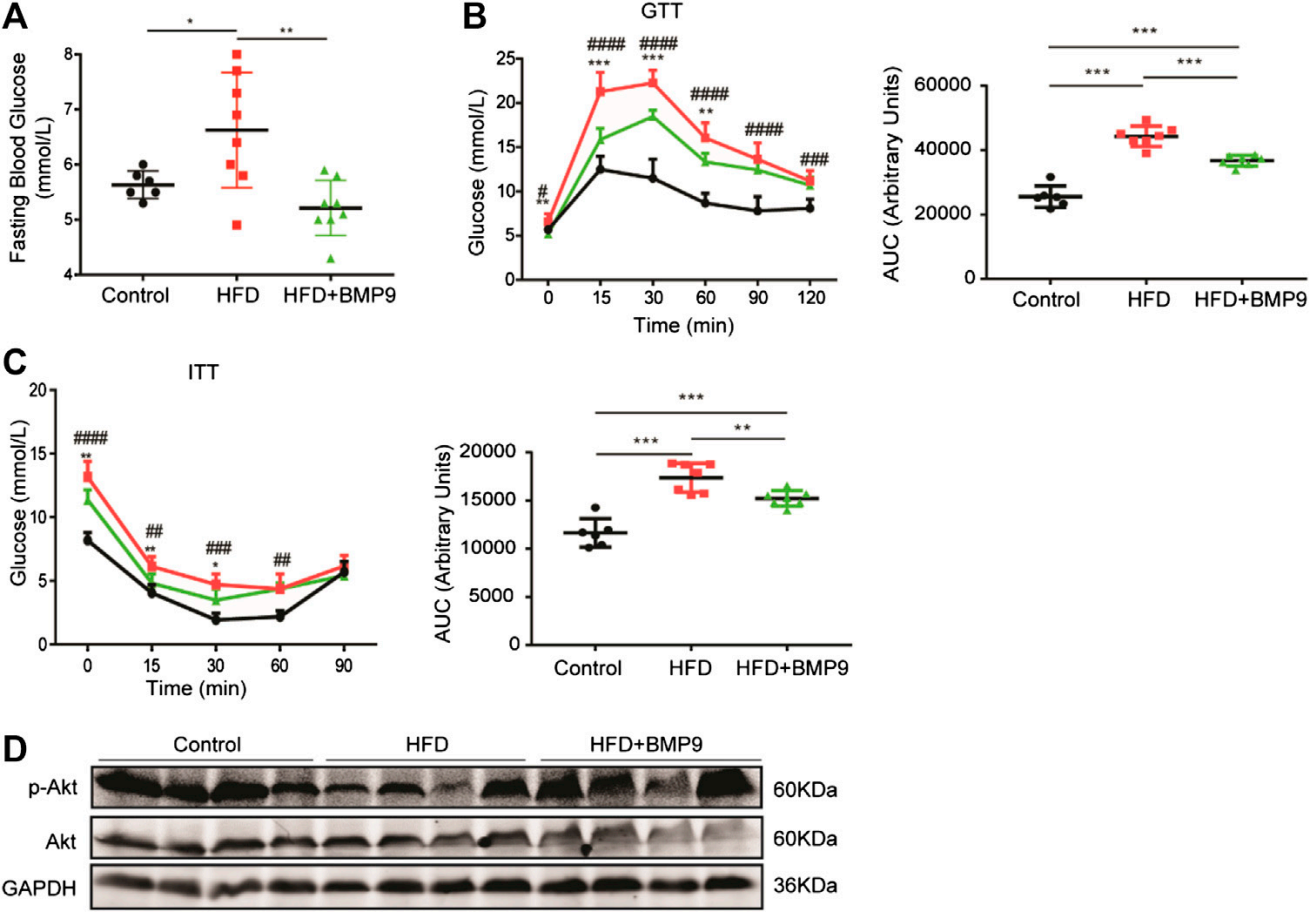
BMP9 modulates glucose metabolism in HFD-induced fatty livers in mice. **(A)** Fasting Blood Glucose levels in different groups (Control *n* = 6; HFD *n* = 8, HFD+BMP9 *n* = 8). **(B)** Blood glucose levels and the area under the curve for glucose during GTT (Control *n* = 6; HFD *n* = 7, HFD+BMP9 *n* = 7). **(C)** Blood glucose levels and the area under the curve for glucose during ITT (Control *n* = 6; HFD *n* = 7, HFD+BMP9 *n* = 7). **(D)** The level of p-Akt in the livers of mice. The data are expressed as the means ± SEM. *p < 0.05, **p < 0.01, ***p < 0.001, ^##^ *p* < 0.01, ^###^ *p* < 0.001, ^####^ *p* < 0.0001.* HFD vs. HFD+BMP9, ^#^HFD vs. Control.

Obesity is often accompanied by abnormal glucose metabolism. Therefore, we further observed the effects of BMP9 on glucose homeostasis by testing fasting blood glucose (FBG) levels, IPGTT and IPITT. As shown in Figure 4A, FBG levels of HFD mice were elevated compared with control mice, and BMP9 treatment decreased FBG levels markedly in HFD-fed mice. Compared to normal mice, HFD mice exhibited a significant impairment in glucose tolerance and induced insulin resistance, as indicated by IPGTT and IPITT, respectively (Figures 4B,C). BMP9 treatment also increased glucose tolerance and decreased insulin resistance in HFD mice. Insulin sensitivity was further evaluated by measuring the levels of phosphorylated Protein Kinase B (p-Akt) in different mouse livers. As shown in Figure 4D, HFD mouse livers had lower levels of p-Akt compared to controls. BMP9 treatment also reversed the levels of p-Akt. Taken together, these data confirmed that BMP9 also improves glucose metabolism in NAFLD mice.

### BMP9 Downregulates Lipid and Glucose Metabolism Genes as Well as Inflammatory Responses in HFD-Induced NFLD in Mice

**FIGURE 5:**
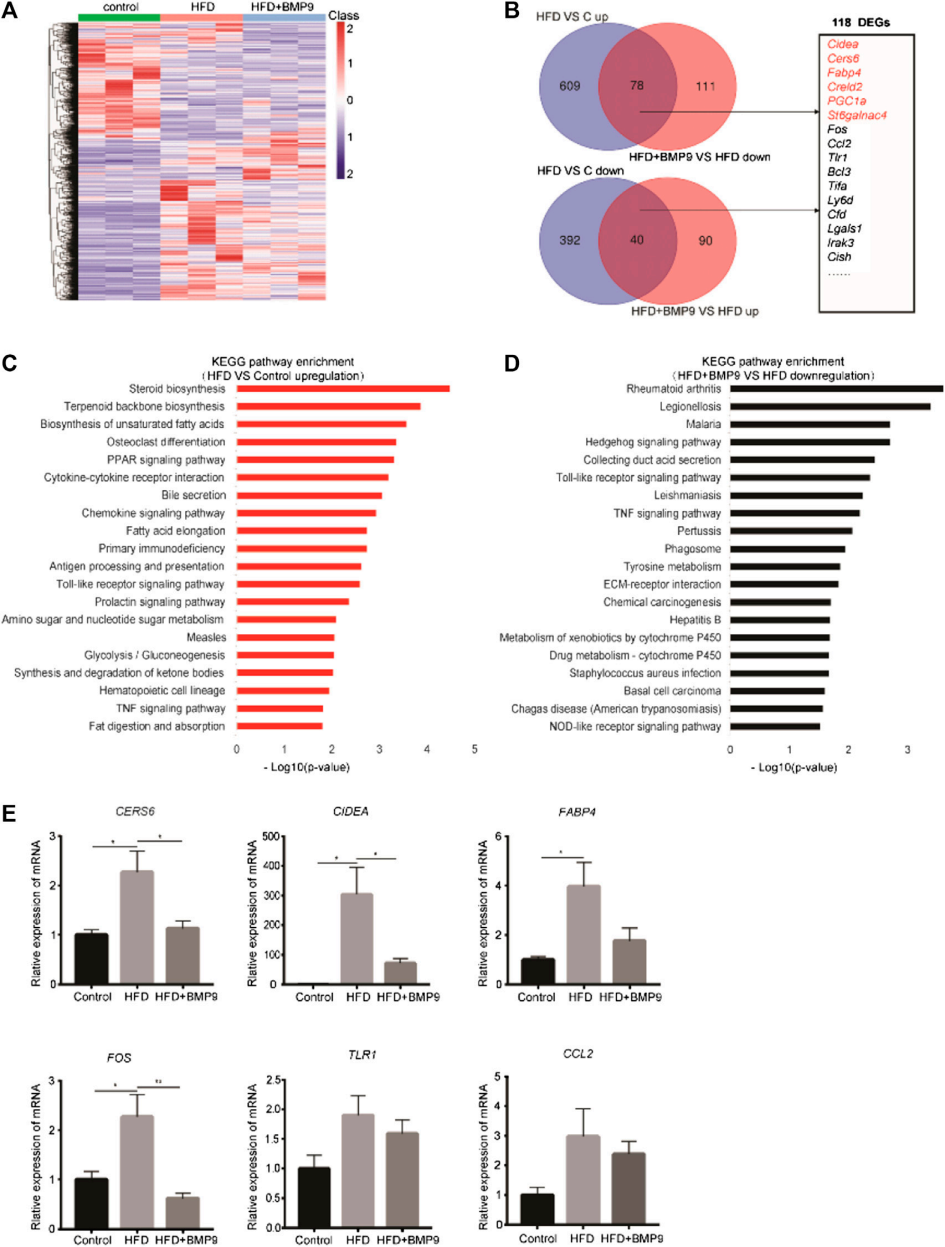
BMP9 reprogrammes transcriptional profiles of HFD-fed mouse livers. **(A)** Hierarchical cluster analysis of all DEGs in the three groups, *n* = 3 in each group. *p* < 0.05, |Log2FC|>1. **(B)** DEGs with opposite trends in HFD vs Control and HFD+BMP9 vs. HFD, and sixteen DEGs associated with lipid metabolism and inflammation are listed. *p* < 0.05, |Log2FC|>1. **(C)** KEGG (Kyoto Encyclopedia of Genes and Genomes) annotation for the upregulated genes between the HFD and Control groups. **(D)** KEGG annotation for the downregulated DEGs between the HFD+BMP9 and HFD groups. **(E)** Validation of some DEGs by qRT-PCR in mouse livers. The data are expressed as the means ± SEM. *p < 0.05, **p < 0.01.

To elucidate the mechanism of BMP9 in alleviating HFD-induced NAFLD, RNA-seq was used to detect alterations in the transcriptional profiles of BMP9-treated HFD mouse livers. As shown in Figure 5A, the heat map shows the overall differentially expressed genes (DEGs) of the livers from three groups of mice. We identified 687 genes that were upregulated and 432 genes that were downregulated in HFD mouse livers vs. control mouse livers. In addition, BMP9 treatment resulted in the downregulation of 189 genes and upregulation of 130 genes in HFD mouse livers (Figure 5B, Supplementary Tables S2–5). Further analysis revealed that BMP9 treatment reversed the expression of 118 genes deregulated in HFD mouse livers, including the genes involved in lipid and glucose metabolism as well as inflammatory and immune response genes (Figure 5B). The top 20 Kyoto Encyclopedia of Genes and Genomes (KEGG) pathways of the upregulated genes in HFD mouse livers vs. control mouse livers are shown in Figure 5C, and the KEGG pathways of downregulated genes are shown in Fig. S2A. Among the top 20 enriched KEGG pathways associated with upregulated DEGs, steroid biosynthesis, PPAR signaling and fatty acid elongation were involved in lipid metabolism; glycolysis/gluconeogenesis was related to glucose metabolism; and Toll-like receptor and TNF signaling pathways were associated with inflammatory and immune response (Figure 5C, Supplementary Table S6–9). Interestingly, the upregulation of Toll-like receptor signaling and TNF signaling by HFD was reversed in the livers of BMP9-treated HFD-fed mice (Figure 5D). The expression of some DEGs involved in lipid and glucose metabolism, including Cers6, Fabp4 and Cidea (Zhou et al., 2012; Furuhashi et al., 2014; Hammerschmidt et al., 2019; Kim et al., 2019), and related to inflammatory responses, including Fos, Tlr1 and Ccl2, were further validated by RT-PCR in three groups of mouse livers (Figure 5E). These data showed that BMP9 alleviated hepatic steatosis by downregulating lipid and glucose metabolism genes as well as by decreasing inflammatory responses in HFD-induced NAFLD mice.

### BMP9 Regulates the Expression of Genes Related to NAFLD by Modulating Chromatin Accessibility

**FIGURE 6:**
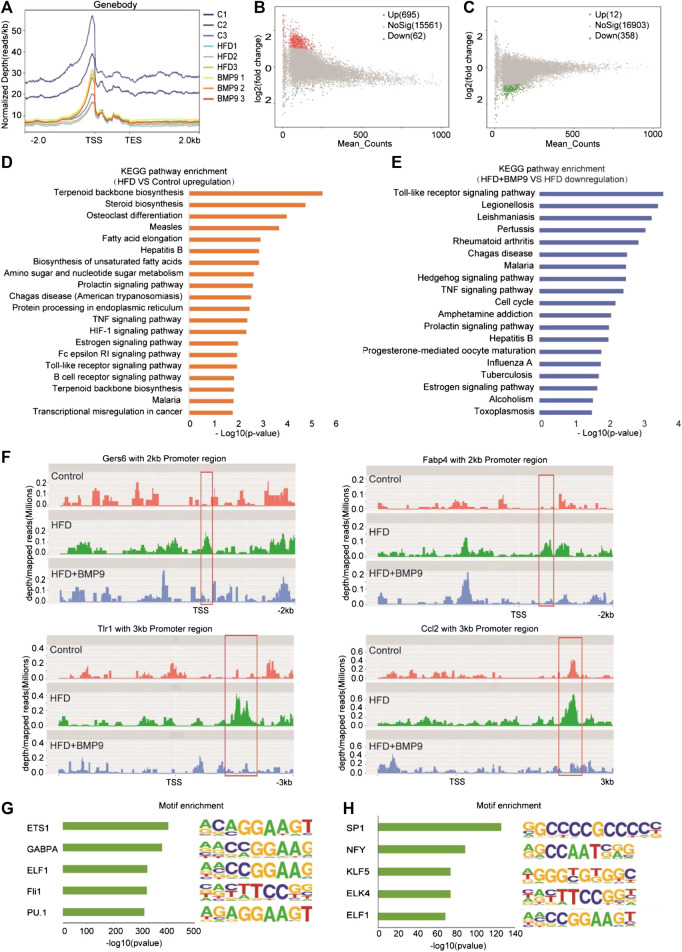
BMP9 changes the chromatin accessibility of HFD-fed mice. **(A)** Chromatin accessibility around the TSS in Control, HFD and BMP9-treated HFD mice. *n* = 3 in each group. **(B)** and **(C)** Scatter plot of the accessibility at each peak in the different groups. **(B)** HFD vs. Control and **(C)** HFD+BMP9 vs. HFD. The red points represent upregulation, and green points represent downregulation. TES: Transcription end site; TSS: Transcription start site. **(D)** and **(E)** Histogram of KEGG (Kyoto Encyclopedia of Genes and Genomes) annotation for the genes with trends in the HFD+BMP9 vs. HFD groups. Orange represents upregulation, and treen represents downregulation. **(F)** Depth mapped reads analysis show signal levels of Cers6, Fabp4, Ccl2 and Tlr1 with 2–3 kb promoter regions in the different groups. **(G)** and **(H)** Motif-enrichment analysis of the downregulated accessible sites and upregulated accessible sites, respectively.

A previous study reported that HFD mouse livers have more open chromatin compared to normal mouse livers, suggesting that chromatin accessibility may play an important role in the progression of NAFLD. Then, we further investigated the effect of BMP9 on chromatin accessibility by ATAC-seq. The ATAC-seq libraries showed that the majority of the insert fragments were small, representing open chromatin between nucleosomes (Supplementary Figure S3A). The accessibility of transcriptional start sites was significantly enriched (Figure 6A, Supplementary Figure S3B), which indicates good ATAC-seq quality. In general, differentially open chromatin regions showed more accessibility in HFD mouse livers vs. controls (Figure 6B), which is consistent with a previous study. BMP9 treatment decreased the openness of chromatin regions in HFD mouse livers (Figure 6C). Integration analysis of RNA-seq and ATAC-seq was further performed. KEGG analysis of the annotated genes associated with the open chromatin regions in HFD mouse livers also identified signaling pathways involved in lipid and glucose metabolism and inflammatory responses (Figure 6D). Consistently, BMP9 treatment downregulated Toll-like receptor signaling and TNF signaling (Figure 6E). Therefore, the integrated analysis of RNA-seq and ATAC-seq further confirmed that BMP9 alleviates inflammatory responses in HFD-fed mouse livers.

To explore the relationship between chromatin accessibility and transcription levels, depth/maps reads of genes were further analysed. The results showed enrichment of ATAC-seq signals at the TSS regions of Cers6, Fabp4, Tlr1 and Ccl2, whose expression was significantly upregulated by RNA-seq, were increased in HFD mice, indicating that these highly transcribed genes showed a more open chromatin landscape in HFD mouse livers than controls (Figure 6H). Further, the increasing ATAC-seq signal levels were decreased by BMP9 treatment. These results indicated that the HFD-induced open chromatin regions play functional roles in HFD-associated transcriptional profile alterations. However, BMP9 reversed the effect of HFD on mouse livers. Overall, BMP9 alleviates NAFLD, at least partly by regulating the landscape of chromatin and decreasing the transcriptomic expression of genes involved in the progression of NAFLD.

To elucidate potential mechanisms of the relationship between transcriptional aberrations and open chromatin regions in HFD mice with or without BMP9 treatment, we investigated DNA transcription factor binding at dysregulated chromatin accessible sites. The 5 most enriched transcription factor motifs in the decreased and increased chromatin accessible regions in BMP9-treated mouse liver vs. PBS-treated mouse liver are shown in Figures 6G and H. In addition, all enriched motifs are listed in Supplementary Tables S10 and S11. Among the top 5 enriched motifs, PU.1 has been demonstrated to play a key role in improving glucose homeostasis and alleviating mouse liver inflammation and hepatic steatosis. Moreover, SP1 and NF-Y increased fatty acid β-oxidation by upregulating carnitine palmitoyl transferase I (CPTI) expression in rat livers. The roles of ETS1, GABPA, ELF1, Fli1, KLF5, ELK4 and ELF1 in NAFLD have rarely been studied. These TFs may be responsible for the regulation of transcriptional profiles. But these were associative results of ATAC-seq data, the exact role of these TFs in the reduction of NAFLD by BMP9 needs further study.

## Discussion

NAFLD is a major healthcare burden worldwide without effective pharmacological therapy. Although multi-factors are involved in the progression of NAFLD, the most important factors are steatosis, insulin resistance and inflammation. In recent years, there has been mounting evidence that BMP9 regulates glucose and lipid metabolism (Miller et al., 2000; Miller et al., 2000; Bi and Ge, 2014; Li et al., 2018; John et al., 2019). However, its role in NAFLD is unclear.

In our study, we found decreased mRNA and protein expression of BMP9 in the livers of HFD-fed mice compared to those of controls. This result was consistent with another previous study (Yang et al., 2019). We also found that BMP9 could: 1) alleviate obesity, liver steatosis and macrophage infiltration; 2) improve glucose tolerance and insulin sensitivity; 3) downregulate genes related to lipid and glucose metabolism, as well as inflammatory responses; 4) and reshape chromatin accessibility in HFD-induced NAFLD mice. Our results suggest that BMP9 plays important roles in lipid and glucose metabolism as well as in inflammatory responses in the progression of NAFLD.

Previous studies have shown that BMP9 can decrease SREBP1 expression by inhibiting liver X receptor response element 1 activity in SREBP1 (Yang et al., 2019). In our study, BMP9 also reduced SREBP1 expression. However, the downregulation of other lipid and glucose metabolism genes, such as Cers6, Cidea and Fabp4, was more significant. Some of these genes have been closely associated with NAFLD.

CerS6 is a C16:0 ceramide synthase, which affects distinct sphingolipid pools. Abrogation of CerS6 protects from obesity and insulin resistance in mice (Hammerschmidt et al., 2019). Kim et al. found that CerS6 expression increased in the steatotic livers of mice fed a high fat diet, and overexpression of Cers6 *in vitro* increased SREBP1 cleavage and lipogenesis (Kim et al., 2019). These studies suggest that Cers6 plays an important role in lipid and glucose metabolism and may be a key factor in the progression of NAFLD. Cidea (cell death-inducing DNA fragmentation factor-alpha-like effector a) has been reported to play critical roles in hepatic steatosis (Zhou et al., 2012). Cidea expression increased in human hepatic steatosis. Overexpression of Cidea aggravated hepatic steatosis, and Cidea deficiency alleviated this effect in mouse livers. Fatty acid binding protein 4 (Fabp4) is a cytoplasmic fatty acid transporter. It is mainly expressed in adipocytes and macrophages and plays a critical role in the progression of insulin resistance (Furuhashi et al., 2014). Targeting Fabp4 with an inhibitor can significantly improve insulin resistance in leptin-deficient *ob/ob* mice (Furuhashi et al., 2007). Dysregulated FABP4 has been associated with obesity and NAFLD (Thumser et al., 2014). In our study, we found that BMP9 could downregulate the expression of Cers6, Cidea and Fabp4. Therefore, we speculated that BMP9 might alleviate hepatic steatosis and improve glucose metabolism by downregulating Cers6, Cidea and Fabp4 expression. Our results showed that BMP9 affects a series of genes related to lipids and glucose. Therefore, we suspected that the effect of BMP9 on lipid and glucose metabolism was broad.

It has been recognized that macrophage-mediated inflammation plays a vital role in the progression of NAFLD (Alisi, A., et al., 2017). Liver macrophages comprise Kupffer cells (resident in the liver) and infiltrating monocytes recruited from the circulation mainly by CCL2/CCR2 signaling (Kazankov et al., 2019). Liver macrophages can be further divided into M1 (pro-inflammatory) and M2 (anti-inflammatory) subtypes, in which M1 is recognized as a key pathogenic factor in the development of NAFLD. In our study, BMP9 could decrease macrophage infiltration and downregulate the expression of Ccl2. Whether BMP9 decreased liver macrophage infiltration by decreasing Ccl2 expression and altered the M1/M2 ratio by inducing macrophage M2 polarization requires further study. Ccl2, Fos and Tlr1 were upregulated in HFD mouse livers compared to controls and downregulated by BMP9 treatment. Moreover, Ccl2, Fos and Tlr1 are enriched in TNF and TLR signaling pathways, which were significantly decreased KEGG pathways in BMP9-treated NAFLD mouse livers. Our results showed that BMP9 downregulated inflammatory genes in HFD mouse livers, such as Tlr, Fos and Ccl2, and alleviated HFD-induced TNF and TLR signaling pathways. According to these data, we speculated that BMP9 might reduce the expression of Ccl2, thereby changing the number or composition of liver macrophages, and ultimately alleviating liver inflammation. BMP9 may be a potential treatment strategy for NAFLD by alleviating liver inflammation. However, the specific mechanism needs further study.

It has been reported that mice with HFD-induced NAFLD also exhibit alterations in chromatin accessibility and gene expression (Leung et al., 2014). Our study verified that chromatin of HFD mouse livers was more open than controls. To explore the mechanism of BMP9 downregulating genes involved in NAFLD, depth mapped reads of these genes were analysed. Lower signal levels were found in TSS regions of Cers6, Fabp4, Ccl2 and Tlr1 in BMP9-treated mouse livers. These changes may have resulted in less TF binding to DNA and less transcriptional activation. Potentially changed transcription factors were enriched by peak diversity between BMP9- treated and PBS-treated HFD mice. Nine TFs (ETS1, SP1, GABPA, NFY, ELF1, ELK4, ELK5, Fil1 and PU.1) were predicted to be key factors in the process of BMP9 alleviating NAFLD. PU.1, SP1 and NFY have been reported to participate in lipid and glucose metabolism. PU.1 knockdown in the liver improved glucose metabolism in dit-induced obese mice (Liu et al., 2020). SP1 and NF-Y upregulate CPTI expression and increase lipid oxidation (Steffen et al., 1999). In our study, BMP9 downregulated PU.1 and upregulated SP1 and NFY. Whether BMP9 alleviate NAFLD by decreasing liver chromatin accessibility and regulating bindings of these TFs to target genes needs deep research. At the same time, the roles of other TFs in NAFLD also merit further study.

**FIGURE 7:**
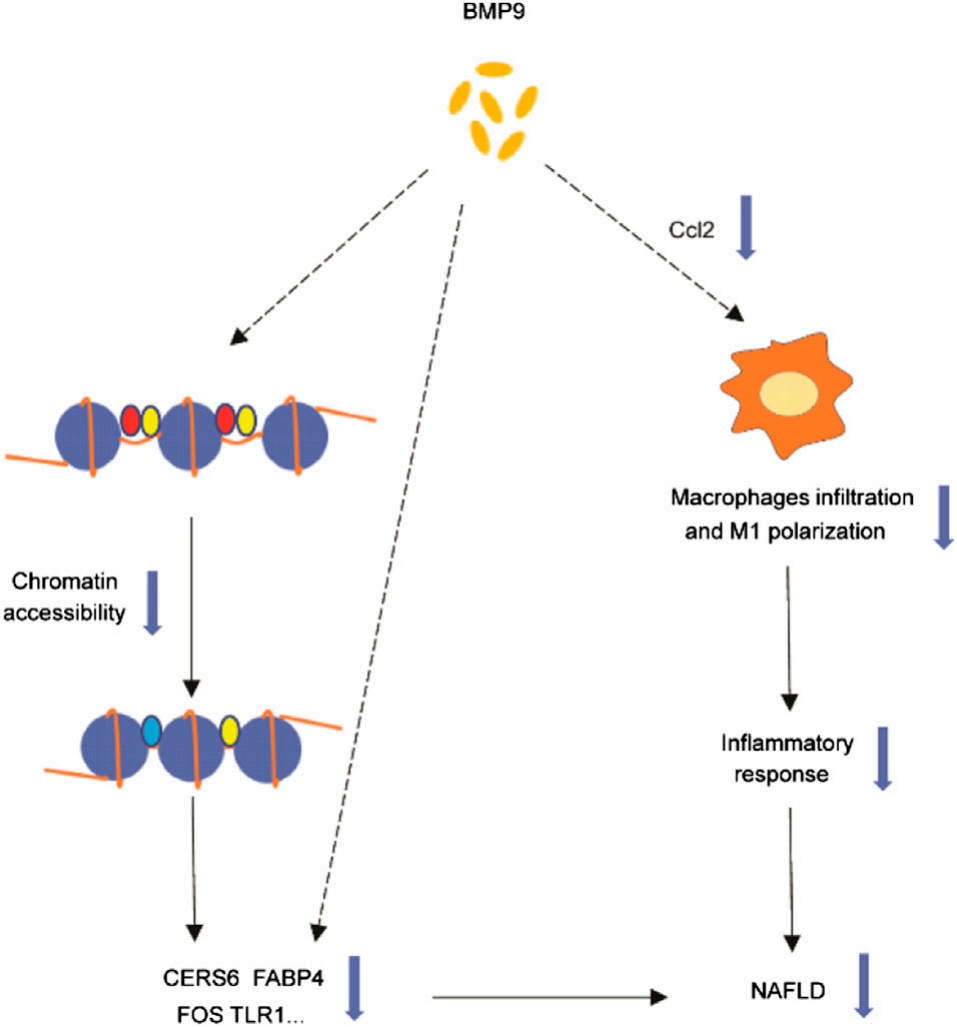
Graphical abstract of our study. BMP9 downregulated the expression of Cers6, Cidea, Fabp4, Ccl2, Tlr1 and Fos related to NAFLD. BMP9 regulates these genes through changes in chromatin accessibility or other mechanisms that needs further study. BMP9 might reduce the expression of Ccl2, thereby changing the number or composition of liver macrophages, and ultimately alleviating liver inflammation. The dotted line indicates that the mechanism is unknown.

In summary, our study found that BMP9 downregulated the expression of Cers6, Cidea, Fabp4, Ccl2, Tlr1 and Fos related to NAFLD. We further confirmed that BMP9 could alleviate hepatic steatosis and insulin resistance in HFD-fed mice. Furthermore, BMP9 could alleviate liver inflammation probably via reducing Ccl2 expression, which has not been previously reported. This suggested that BMP9 ameliorates NAFLD not only by improving lipid and glucose metabolism but also via decreasing macrophage infiltration and inflammation in mouse livers. Our study suggests that the role of BMP9 in NAFLD may be multi-faceted, involving more than just lipid and glucose metabolism. However, it’s not clear whether BMP9 alleviates NAFLD through direct action on the liver or the results of improved glucose and lipid metabolism. And it’s also unknown whether BMP9 regulates these genes only through changes in chromatin accessibility, or whether there are other mechanisms. Is Ccl2 a key factor for BMP9 to reduce liver inflammation? Therefore, more research is needed in the future. The disadvantage of the study lies in the lack of further experiments to explore mechanisms and verify. However, based on our results, BMP9 still has potential in the treatment of NAFLD. The graphical abstract of our study were showed in Figure 7

### Future Perspective

Study of the mechanism that BMP9 alleviates NAFLD still has a long way to go. The database was enriched by our epigenomic research in this study. We speculate that the effect of BMP9 on inflammatory response is more significantly and further study will be done in the future on the mechanism of BMP9 and NAFLD.

## Data Availability Statement

The datasets presented in this study can be found in online repositories. The names of the repository/repositories and accession numbers can be found in the article/Supplementary Material.

## Ethics Statement

The animal study was reviewed and approved by the Scientific Investigation Board of the Second Military Medical University.

## Author Contributions

LZ and XZ obtained funding. Q-JS and LZ designed the study and wrote the manuscript. Q-JS, S-QL, JJ, WW, J-LL, and Y-LC performed the experiments. Q-JS and L-YC contributed to the analysis and interpretation of data. Q-JS performed the statistical analysis. C-KH provided material support. All authors revised the manuscript.

## Funding

This work was supported by Science and Technology Development Fund of Shanghai Pudong New Area, PKJ2016-Y62 and the Top-Level Clinical Discipline Project of Shanghai Pudong (PWYgf2018-04), and Technology Development Project of Pudong Science, Technology and Economic Commission of Shanghai (Grant No.PKJ2017-Y17). The authors have no other relevant affiliations or financial involvement with any organization or entity with a financial interest in or financial conflict with the subject matter or materials discussed in the manuscript apart from those disclosed.

## Conflict of Interest

The authors declare that the research was conducted in the absence of any commercial or financial relationships that could be construed as a potential conflict of interest.
